# Parents' attitudes and behaviours towards recommended vaccinations in Sicily, Italy

**DOI:** 10.1186/1471-2458-11-305

**Published:** 2011-05-12

**Authors:** Maria Anna Coniglio, Marco Platania, Donatella Privitera, Giuseppe Giammanco, Sarina Pignato

**Affiliations:** 1 Department of Hygiene and Public Health "G. F. Ingrassia", via Santa Sofia 87, 95123, University of Catania, Catania, Italy; 2 Department of Formative Processes, via Biblioteca 4, 95124, University of Catania, Catania, Italy

## Abstract

**Background:**

Since a long time, Italy has maintained a dual system to administer childhood immunisations, that is a certain number of mandatory vaccinations and a number of recommended vaccinations. The study aimed to explore the issues surrounding parental acceptance or non-acceptance of the recommended vaccinations for children.

**Methods:**

Parents of children aged 3-5 years of day-care centres in Sicily were asked to fill out an anonymous questionnaire. Determinants of the attitude towards recommended vaccinations and social influence on the decision-making process were assessed using logistic regression analysis.

**Results:**

Of the 1,500 selected parents, 81.0% participated in the study. Prior to the survey, the majority of children (97.6%) received recommended vaccines. Most parents (74.4%) received information about vaccinations from Family Paediatricians, showed a good knowledge about the side effects of the vaccines (73.1%), did not worry about their potential dangerousness (53.0%) and would have accepted their children to be vaccinated even if it was not required for day care (84.1%). The majority (79.9%) were not disposed to follow the advises of the anti-vaccination movements. Parents' background characteristics, sources of information and social influence were not significantly associated with parental acceptance of recommended vaccines for childhood.

**Conclusions:**

This study suggests that health information by Family Paediatricians is significantly associated with parental acceptance of recommended vaccinations.

## Background

Since a long time, Italy has maintained a dual system to administer childhood immunisations, that is a certain number of mandatory vaccinations and a number of recommended vaccinations. At present, mandatory vaccinations are four: inactivated polio (IPV), diphtheria-tetanus (DT), and viral hepatitis B (HBV). Vaccinations against measles, mumps and rubella (MMR), pertussis, varicella, pneumococcal disease and *Haemophilus influenzae *b (Hib) are nationwide included among recommended immunisations. Until 1995 no organic policy of offer of recommended vaccinations existed. Since that year, a Circular Letter of the Ministry of Health (no. 13, 6 June 1995) established that all the Italian regions should actively offer MMR and acellular pertussis vaccines free of charge. The National Health Plan 1998-2000 set a 95% coverage goals for both mandatory and recommended vaccinations of childhood. In addition to these regulatory measures, many new combination vaccines were introduced on the market since 1999, and hexavalent products including all antigens to be administered in the first year of life (DT, acellular pertussis, IPV, HBV, Hib) have been available since 2001. In 2002, all vaccinations included in the national schedule were placed on the list of "essential health interventions" that all regions must provide free of charge within the National Health Service (NHS).

Among the 20 Italian regions, Sicily has one of the better immunisation coverage towards the recommended vaccinations for out of preschools children: 95% for pertussis and Hib, 87.5% for varicella, 86.9% for MMR [1,2]. Universal varicella vaccination was added to the standard childhood vaccination programme in January 2003 and was actively offered free of charge to all children at 15 months of age and to all susceptible adolescents in their 12th year of age, at the time of the MMR vaccination in order to improve parents compliance. Moreover, in 2005 Sicily took priority to the first goal of the Italian National Prevention Plan 2005 - realization of a computerized Register of Vaccinations - which was considered propaedeutic and essential for the improvement of the recommended immunization coverage. In particular, the most important measures taken by the Sicilian region were: *i) *improvement of the heptavalent vaccination coverage towards pneumococcal disease, *ii) *elaboration of a Regional Vaccination Planner, *iii) *surveillance and control of measles and varicella, *iv) *improvement of the MMR vaccination coverage.

Nevertheless, for an efficacious and safe vaccine to prove its potential, it must be accepted, beyond the national health authorities, by health care workers and, last but not least, by the population at large [3-5]. Clearly, the decision to accept or forgo vaccination is not a simple matter.

In an effort to understand this decision-making process more clearly, this study was undertaken: *(i) *to explore the issues surrounding parental acceptance or non-acceptance of the recommended vaccinations; *(ii) *to verify whether in a health maintenance organization such as the Italian NHS, where comprehensive vaccine coverage is emphasized, factors traditionally considered influent in the decision making process are significantly associated with parental acceptance of recommended vaccines for childhood. Finally, a sample of Sicilian preschool children was chosen for two main reasons: (*i*) among all the Italian regions, Sicily has one of the highest coverage rates of recommended immunisation for out of preschool and preschool children; (*ii*) free vaccination is available in Sicily for recommended vaccines in out of preschool and preschool children.

## Methods

### The sample and the questionnaire

The study has been approved by the Ethics Committee of the "Azienda Ospedaliero-Universitaria Policlinico-Vittorio Emanuele, Catania". The study was conducted between October and December 2008 among parents of children aged between 3 and 5 years who attended public day care centres near Catania, a town of 308.400 inhabitants, located in the east coast of Sicily with similar vaccine uptake as in Sicily as a whole. Day care centres location was divided into 2 groups: central areas and peripheral areas. Eight out 80 public day care centres (10%) were randomly selected. A systematic sample [6] of 1,500 parents with at least 3 members in nuclear family was selected.

In order to explore factors affecting parents' action with respect to the recommended childhood vaccinations, we conducted a questionnaire study. In particular, as shown in Table 1, a self-administered questionnaire evaluated parents' demography and their children's vaccination status according to the Italian scheme for the recommended vaccines. Also, it evaluated perceived individual and population protection by recommended vaccinations, and the attitude towards compliance with recommended vaccinations that are currently available, but not included in the program for all children. Finally, it explored social influence of persons (e.g., their family paediatrician or friends), mass or press media and websites, as well as their opinion about the anti-vaccination movements. Parents were given 20 minutes to complete the questionnaires, which were returned directly to the researchers in a prepared envelope to protect anonymity.

**Table 1:** Explored variables

*Parents' background variables*	• Var. 1: Parental status • Var. 2: Age • Var. 3: Education level • Var. 4: Number of children per family
*Evaluation of parents' attitudes, knowledge and beliefs concerning recommended vaccines*	
Item 1	Children's vaccination status
Item 2	Decision to forgo recommended vaccinations
Item 3	Physician recommendations prior to vaccinations
Item 4	Sources of information on recommended vaccinations
Item 5	Distinction between mandatory and recommended vaccinations
Item 6	Knowledge on mandatory and recommended vaccinations
Item 7	Perceived recommended vaccinations usefulness
Item 8	Perceived recommended vaccinations effectiveness
Item 9	Social influence on decision-making process
Item 10	Source of social influence
Item 11	Knowledge of the existence of anti-vaccination movements
Item 12	Agreement with anti-vaccination movements
Item 13	Opinion about vaccinations
Item 14	Opinion about the anti-vaccination movements

### Statistical analyses

Preliminary analyses consisted of examining descriptive analyses (e.g., numbers and frequency distributions for quantitative and qualitative variables). Subsequently, the component variables in the decision-making process were defined. Logistic regression was used to test the hypothesized relationships among the set of background variables, the set of decision-making process variables, and the agreement variable. These variables included parents' perceived recommended vaccinations effectiveness. The independent variables were factors reflecting parents' background characteristics such as age, education level and factors influencing the decision-making process (e.g., sources of information on vaccinations and social influence on decision-making process).

## Results

### Descriptive analyses

Eighty-one percent (n = 1,218) of the selected parents (n = 1,500) participated in the study, with similar distribution in the two areas (48.8% in the central area and 51.2% in the peripheral area). Ninety-three percent of respondents were mothers (n = 1,133), and seven percent were fathers (n = 85). Parents' age range was 31 to 40 years. More than 50.0% of the respondents stated a high education level (college and university graduates together accounted for 56.1% of the sample). Only 4.1% reported more than 3 children per family. Prior to the survey, the vast majority of children (97.6%) were vaccinated according to the national recommended vaccination scheme.

As shown in Table 2, at the time of the survey most parents (74.4%) had received information about recommended childhood vaccinations from their Family Paediatrician (FP). Other sources of information were press (13.2%) or mass media (8.5%), and websites (3.3%). More than seventy percent (73.1%) of the respondents showed a very good knowledge about the possible side effects of childhood vaccinations and more than fifty percent (53.0%) did not worry about the potential dangerousness of the vaccines, identifying the prevention of discomfort and complications of the diseases as the primary benefit of vaccinations. Moreover, the majority of the respondents (84.1%) stated that they would have accepted their children to be vaccinated even if it was not required for day care or preschool, being aware that both mandatory and recommended vaccinations are necessary for their children. A relatively low but not negligible percentage of parents knew the existence of anti-vaccination movements (16.8%) and admitted to agree with their ideology (10.4%). Nonetheless, prior to the survey, the vast majority of these parents (96.1%) decided to vaccinate their children according to the national recommended vaccination scheme. Finally, most parents (79.9%) considered the anti-vaccination movements as alternative information tools but were not disposed to follow their advises. In fact, a high percentage of these parents (98.2%) decided to vaccinate their children with recommended vaccines.

**Table 2:** Awareness, attitudes and beliefs towards vaccines in the study sample

Awareness		
*Sources of information about recommended vaccinations*	N°	%
Family Paediatrician	906	74.4
Leaflets/Magazines	160	13.2
Television	103	8.5
Websites	40	3.3
Other	9	0.6
Total	1,218	100.0
*Knowledge of the distinction between mandatory and recommended vaccinations*		
Good knowledge	1,062	87.2
Bad knowledge	156	12.8
Total	1,218	100.0
*Possible side effects of vaccines*		
Very good knowledge	890	73.1
Good knowledge	88	19.7
Bad knowledge	240	7.2
Total	1,218	100.0
**Attitudes and beliefs**		

*Opinion about vaccinations*		
Vaccines are always dangerous	12	1.0
Vaccines are sometimes dangerous	493	40.5
Vaccines are not dangerous	645	53.0
Do not know	68	5.5
Total	1,218	100.0
*Vaccination acceptance even if it would be not required*		
Yes	1,024	84.1
No	142	11.7
Do not know	52	4.2
Total	1,218	100.0
*Knowledge of anti-vaccination movements*		
Yes	205	16.8
No	1,013	83.2
Total	1,218	100.0
*Opinion about anti-vaccination movements*		
They should be forbidden	39	3.2
They should be promoted	127	10.4
They are alternative information tools	973	79.9
Do not know	79	6.5
Total	1,218	100.0

### Logistic regression analysis

Before the analyses were carried out, the presence of multicollinearity between independent variables (items 2, 3, 4 and 9) was assessed using the correlation matrix. As |r| ≤ 0.7 for all the pair correlations, it was assumed that the model was not affected by multicollinearity. Moreover, given the high number of phenomena whose influence on the dependent variable needed to be assessed, the logistic regression function was calculated with the stepwise procedure. The forward selection procedure was adopted, that is to say the elaboration algorithm that selects step by step the variables to be included in the model. The Hosmer-Lemershow test showed that the model adequately fitted the data. Further assessment of the model was carried out with the pseudo R-squared statistic (Table 3). On a scale of 0 to 1, high values for R-squared indicated that much of the variation of the dependent variables could be explained by the model. The elaboration model showed a low significance (< 1%) of the Cox-Snell and Nagelkerke R Squares, indicating that parents' attitudes and behaviours about recommended vaccinations cannot be predicted from the linear combination of the independent variables considered. For this reason, it was decided to stop the elaboration.

**Table 3:** Model summary

Step	-2 Log likelihood	Cox & Snell R Square	Nagelkerke R Square
1	800,145(a)	,016	,030
2	789,882(b)	,025	,047
3	783,785(b)	,030	,057
4	776,584(b)	,036	,069
5	771,834(b)	,040	,077
6	767,556(b)	,044	,084
7	763,633(b)	,047	,091

## Discussion

The purpose of this study was to define and measure several component parts of parents' decision-making process about the recommended vaccinations for children. After identifying the various components, the intent was to determine if some background variables, specific factors influencing the decision-making process (e.g., social status and sources of information about vaccinations), and parents' perceived vaccinations effectiveness were useful in predicting whether parents were likely to have their children vaccinated. A sample of Sicilian parents was chosen because, among all the Italian regions, Sicily has one of the highest coverage rates of recommended immunisation.

Several interesting findings were revealed. First of all, the percentage of parents' positive attitudes towards vaccinations was exceptionally high compared with other similar studies [7-9]. This finding could be linked to the fact that in our study parents showed very good knowledge about the side effects of vaccines and, subsequently, deep awareness of their safety. The explanation for reports of great knowledge and deep awareness could be the trust the parents had in their FP (prior to the survey 74.4% received information from the FP and 97.6% submitted their children to the recommended vaccinations). Secondly, prior to the survey, the vast majority of children (97.6%) were vaccinated according to the national recommended vaccination scheme. This data could be noteworthy because in Italy children can be enrolled in a preschool even if not vaccinated for recommended vaccines.

These data probably could be useful to demonstrate that FP offers a unique surveillance opportunity. In fact, every child in Italy is registered free of charge with a FP from birth until 14 years of age within the NHS. Thus, each FP has a precise paediatric population under his care (800-1000 children) and his public duty includes routine control visits that are perfect opportunities for vaccination offer, disease control and surveillance. Moreover, these findings confirm the results from other studies which indicated that the most frequent factor influencing parents' decision about childhood vaccinations was advice from doctors [10-13].

Another interesting finding is related to the evaluation of predictive factors useful in explaining possible negative attitudes towards recommended vaccinations. Differently from other similar studies [5,12,14-16], our findings showed a lack of correlation among the decision to have their children vaccinated and factors traditionally considered important in influencing the parents' decision making process: parents' socio-demographic characteristics (education level, age and sex), presence of other children in the nuclear family, possible influence of press, mass or web media.

Nevertheless, potential biases should be considered before generalizing these results to all parents of Italian young children. The most important possible bias is that the parents who agreed to participate in the study may be those who were most in favour of vaccinations and therefore the most inclined to vaccinate their children with recommended vaccinations. This bias is unavoidable and, although we are not able to provide information on refusals, we think that its influence would be the same in the rest of Italy.

One other bias could be linked to the fact that we do not really know whether children were vaccinated or not with recommended vaccines. In our opinion the effect of this potential bias would have been minimal because the parents completed a strictly anonymous self-administered questionnaire that they returned directly to the researchers in a prepared envelope to protect anonymity. Secondly, official data indicates that in Sicily recommended vaccination rates are more than 80.0% [1].

Another limit of the study is also due to the fact that it did not collect information on religion and parents' level of income. It has been demonstrated that in many countries, due to the lack of official recommendation and of reimbursement, these variables have a strong influence on the acceptance of vaccines [5,17]. Although this information would probably have been useful to better describe the response pattern, we do not believe that this fact influenced our results first of all because the Italian population is quite homogeneous from a religious point of view (in 2006, 95.8% of Italian people were Catholics), and secondly because, differently from other countries, in Italy economics is not a significant barrier to vaccinations because of the free access to health care and, subsequently, to the recommended vaccinations.

Despite potential biases and limits, our results suggest that modifiable determinants for a negative attitude to comply with recommended vaccinations could probably be based on a lack of specific knowledge. In fact, there is still uncertainty surrounding the safety of recommended vaccines. This barrier to vaccinations might be overcome firstly by improving health education of parents in the vaccination programs. Interventions should emphasise the benefits of combining antigens, such as less pain for the child and fewer vaccination visits. Parents may also need information about how recommended vaccines work in order to avoid misconceptions over safety.

Clearly, the NHS needs to do more in informing people about the process of immunisation in order to allay fears (e.g., information in the antenatal period may enable parents to make more informed decisions about recommended vaccinations). In fact, the majority of parents in the present study had information prior to their child's immunisation from their FP. Nevertheless, other sources of information were press or mass media, and websites. Thus, it is still important to ensure that parents have the information to make the informed decisions they desire.

The finding that parents would vaccinate their children even if it would be not required is encouraging because it could indicate a social responsibility. However, parental uncertainty about the need for recommended vaccines is a matter requiring attention. Thus, efforts to improve uptake by FP could focus on community benefits.

Finally, in Italy, parents of preschoolers generally receive an invitation to attend, with no information about the vaccines and the diseases they protect against. Further research by this team is underway to examine parental satisfaction with this and many other aspects of the childhood immunisation service.

## Conclusions

Up to now, the public perception of vaccines has worldwide been influenced by fear of diseases or, more recently, fear of vaccines once the diseases in question have disappeared thanks to vaccinations. It is well known that when vaccination providers are actively involved in information about vaccinations, parents are more convinced about the advantages of the vaccines to prevent serious complications. Nonetheless, although the present study identified general acceptance of the recommended immunisation programme, surely because the majority of parents did place trust in the advice of FP, there is still uncertainty surrounding the safety of recommended vaccines. This implies that when discussing the vaccines with parents, health care providers in general, and FP in particular, should place emphasis on promoting the idea that recommended vaccines will keep their children healthy, are safe and are effective in protecting most children. Moreover, in clinical practice, it is imperative to make clear that the risks of severe illness from diseases preventable by vaccines are far greater than the risks of vaccination.

To conclude, this study suggests that in a health maintenance organization such as the Italian NHS, where comprehensive vaccine coverage is emphasized, factors traditionally considered influent in the decision making process are not significantly associated with parental acceptance of recommended vaccines for childhood. It is also possible that in other countries the absence of reimbursement may affect some parents' decision, due to the cost, and therefore, this may lead to social inequality in coverage immunisation. Thus, further studies in other countries with similar vaccine uptake as in Italy should be done to validate what has been found in this study. Exploration of factors associated with other areas (e.g., how parents' attitudes change over the preschool years and how to develop ways of addressing common uncertainties about immunisation, including the safety of combining antigens and the need for boosters) would create an interesting comparison.

## Competing interests

The authors declare that they have no competing interests.

## Authors' contributions

MAC carried out the interpretation of the data, participated in the coordination of the study and drafted the manuscript. MP and DP carried out the analysis and the management of data. GG and SP contributed to the interpretation and revised the manuscript critically. All authors read and approved the final manuscript.

## Pre-publication history

The pre-publication history for this paper can be accessed here:

http://www.biomedcentral.com/1471-2458/11/305/prepub

## References

[B1] Sammarco S Ciriminna S Casuccio N Pinella V Iacono F Cuccia M Stella G Mollica G Blangiardi F Ferrera G Casella G Canzonieri G Copertura vaccinale e andamento epidemiologico del morbillo in Sicilia BEN Notiziario ISS 2005 http://www.epicentro.iss.it/ben/2005/giugno/1.htm

[B2] Giammanco G Ciriminna S Barberi I Titone L Lo Giudice M Biasio LR Universal varicella vaccination in the Italian paediatric population: rapid uptake of the vaccination programme and morbidity trends over five years Euro Surveill 2009 14 http://www.eurosurveillance.org/ViewArticle.aspx?ArticleId=19321 pii = 19321 19728978

[B3] Niederhauser VP Baruffi G Heck R Parental decision-making for the varicella vaccine J Pediatr Health Care 2001 15 236 243 11562641 10.1067/mph.2001.114848

[B4] Hak E Schonbeck Y De Melker H Van Essen GA Sanders EAM Negative attitude of highly educated parents and health care workers towards future vaccinations in the Dutch childhood vaccination program Vaccine 2005 24 3103 3107 10.1016/j.vaccine.2005.01.074 15837208

[B5] Austin H Campion-Smith C Thomas S Ward W Parents' difficulties with decisions about childhood immunisation Community Pract 2008 81 32 35 18853886

[B6] Kirsh L Survey sampling 1965 Chichester, John Wiley & Sons

[B7] Fredrickson DD Davis TC Arnould CL Kennen EM Hurniston SG Cross JT Bocchini JA Jr Childhood immunization refusal: provider and parent perceptions Fam Med 2004 36 431 439 15181556

[B8] Salmon DA Moulton LH Omer SB deHart MP Stokley S Halsey NA Factors associated with refusal of childhood vaccines among parents of school-aged children Arch Pediatr Adolesc Med 2005 159 470 476 10.1001/archpedi.159.5.470 15867122

[B9] Adler A Herring E Babilsky H Gazala E Cohen A Levy I Parent-dependent barriers to varicella immunization in Israel: the importance of adequate information Acta Paediatr 2007 96 428 431 10.1111/j.1651-2227.2007.00118.x 17407471

[B10] Freeman VA Freed GL Parental knowledge, attitudes and demand regarding a vaccine to prevent varicella Am J Prev Med 1999 17 153 155 10.1016/S0749-3797(99)00063-X 10490061

[B11] Taylor JA Newman RD Parental attitudes toward varicella vaccination. The Puget Sound Pediatric Research Network Arch Pediatr Adolesc Med 2000 154 302 306 10710032 10.1001/archpedi.154.3.302

[B12] Heininger U An internet-based survey on parental attitudes towards immunization Vaccine 2006 24 6351 6355 10.1016/j.vaccine.2006.05.029 16784799

[B13] Dubé E De Wals P Gilca V Boulianne N Ouakki M Lavoie F Bradet R New vaccines offering a larger spectrum of protection against acute otitis media: will parents be willing to have their children immunized? Int J Pediatr Otorhinolaryngol 2009 73 987 981 10.1016/j.ijporl.2009.03.022 19423171

[B14] Davies P Chapman S Leask J Antivaccination activists on the world wide web Arch Dis Child 2002 87 22 25 10.1136/adc.87.1.22 12089115 PMC1751143

[B15] Luman ET McCauley MM Shefer A Chu SY Maternal characteristics associated with vaccination of young children Pediatrics 2003 111 1215 1218 12728141

[B16] Carrasco-Garrido P de Miguel AG Barrera VH Jiménez-Garcìa R Knowledge of Spanish parents about their children's vaccination during the decade 1993-2003 Hum Vaccin 2007 3 212 216 10.4161/hv.3.5.4500 17786036

[B17] Allaert FA Blanc A Megard Y Bertand I Parents' attitudes towards varicella vaccination acceptance in France and Germany: effect of vaccine recommendation and reimbursement (a survey) J Public Health 2009 17 71 76 10.1007/s10389-008-0218-5

